# Effect of Marine-Derived n-3 Polyunsaturated Fatty Acids on Major Eicosanoids: A Systematic Review and Meta-Analysis from 18 Randomized Controlled Trials

**DOI:** 10.1371/journal.pone.0147351

**Published:** 2016-01-25

**Authors:** Jiajing Jiang, Kelei Li, Fenglei Wang, Bo Yang, Yuanqing Fu, Jusheng Zheng, Duo Li

**Affiliations:** 1 Department of Food Science and Nutrition, Zhejiang University, Hangzhou, China; 2 APCNS Centre of Nutrition and Food Safety, Hangzhou, China; University of Southampton School of Medicine, UNITED KINGDOM

## Abstract

**Background:**

Marine-derived n-3 polyunsaturated fatty acids (PUFA) may have a beneficial effect on inflammation via lowering pro-inflammatory eicosanoid concentrations. We aimed to assess the effect of marine-derived n-3 PUFA on prostaglandin E_2_ (PGE_2_), thromboxane B_2_ (TXB_2_), and leukotriene B_4_ (LTB_4_) through systematic review and meta-analysis of randomized controlled trials.

**Method and Findings:**

A structured search strategy on PubMed, Web of Science and Cochrane up to November 2015 was undertaken in this meta-analysis. Standard mean difference was used to calculate the effect size of marine-derived n-3 PUFA on PGE_2_, TXB_2_ and LTB_4_ in a random-effect model. A total of 18 RCTs with 826 subjects were included in this systematic review and meta-analysis. Supplementation of marine-derived n-3 PUFA significantly decreased concentrations of TXB_2_ in serum/plasma in subjects with high risk of cardiovascular diseases (SMD:-1.26; 95% CI: -1.65, -0.86) and LTB_4_ in neutrophils in unhealthy subjects (subjects with non-autoimmune chronic diseases or auto-immune diseases) (SMD:-0.59: 95% CI: -1.02, -0.16). Subgroup analyses showed a significant reduction of LTB_4_ in subjects with rheumatoid arthritis (SMD: -0.83; 95% CI: -1.37, -0.29), but not in non-autoimmune chronic disease patients (SMD: -0.33; 95% CI: -0.97, 0.31). No significant publication bias was shown in the meta-analysis.

**Conclusions:**

Marine-derived n-3 PUFA had a beneficial effect on reducing the concentration of TXB_2_ in blood of subjects with high risk of CVD as well as LTB_4_ in neutrophils in unhealthy subjects, and that subjects with RA showed lower LTB_4_ content with supplementation of marine-derived n-3 PUFA.

## Introduction

Previous studies have shown that inflammation plays a significant role in a number of widespread and destructive chronic diseases, including autoimmune diseases such as rheumatoid arthritis (RA) [1] and non-autoimmune chronic diseases including obesity and insulin resistance [2], cardiovascular disease (CVD) [3] and several neurodegenerative diseases such as Alzheimer’s disease [4]. Several arachidonic acid (AA) -derived eicosanoids exert their significant influence on the inflammatory response. Prostaglandin E_2_ (PGE_2_) is involved in the classic signs of inflammation and possesses both pro-inflammatory and anti-inflammatory actions [5]; thromboxane A_2_ (TXA_2_) (precursor of TXB_2_), formed by platelets, macrophages and poly-morphonuclear leukocytes (PMNs), can induce vasoconstriction and promotes aggregation of platelets as well as adhesiveness of PMNs [6]; leukotriene B_4_ (LTB_4_) can not only increase vascular permeability and enhance local blood flow by stimulating neutrophil secretion [7], but also stimulate other inflammatory substances.

Previous studies have shown that increased intake of n-3 polyunsaturated fatty acids (PUFA), especially marine-derived n-3 PUFAs (eicosapentaenoic acid [EPA] docosapentaenoic acid [DPA], and docosahexaenoic acid [DHA]), are beneficial for coronary heart disease [8], metabolic syndrome [9] and Alzheimer’s disease [10]. Previous evidence has suggested that the positive protective impact of n-3 PUFA on these diseases may be attributed to the anti-inflammatory function, including lowering of blood eicosanoids [11, 12]. However, the effects of n-3 PUFA on AA-derived major eicosanoids still remains controversial: several studies suggested that marine-derived n-3 PUFA resulted in decreased PGE_2_ [13], TXB_2_ [14] and LTB_4_ [15], while increased PGE_2_ [16] and TXB_2_ [17] in response to n-3 PUFA were also found in some other studies. There has been no systematic review and meta-analysis conducted to summarize the available evidence of the effects of n-3 PUFA on major eicosanoids. Therefore, we conducted a systematic review and meta-analysis of randomized controlled trials (RCTs) to assess the effect of marine-derived n-3 PUFA on AA-derived major eicosanoids (PGE_2,_ TXA_2_/TXB_2_ and LTB_4_).

## Materials and Methods

### Search strategy and study selection

To identify randomized controlled studies involving the effects of marine-derived n-3 polyunsaturated fatty acids on major AA-derived eicosanoids (PGE_2_, TXA_2_/TXB_2_, and LTB_4_), we searched three electronic databases, PubMed, Web of Science and Cochrane Library, up to November 2015 for studies of humans published in all languages. We used the following key words for the literature search: (“fish oil” OR “seafood” OR “eicosapentaenoic acid” OR “docosahexaenoic acid” OR “docosapentaenoic acid” OR “n-3 PUFA”) AND (“eicosanoids” OR “prostaglandin” OR “thromboxane” OR “leukotriene”). References of included studies were also reviewed to identify potential publications. We contacted authors for the detailed information of primary studies when possible.

Search strategy and study selection were undertaken independently by two investigators, with discrepancies resolved through group discussion. Inclusion criteria were: randomized controlled trial design including parallel and crossover design; the exposure of interest was marine-derived n-3 PUFA such as EPA and/or DHA on PGE_2_ or TXB_2_ or LTB_4_; LTB_4_ was determined in neutrophils by HPLC and PGE_2_ and TXB_2_ were measured by Radioimmunoassay or ELISA (enzyme-linked-immunosorbent assay). Studies were excluded if: subjects were diagnosed with acute disease or the duration was less than two weeks; there was dietary invention such as marine fish or others; there were other additional interventions apart from n-3 PUFA such as exercise and anti-inflammatory drugs compared with control group; data indispensable for a meta-analysis were not reported and still unavailable after contacting authors; studies with a crossover design which did not report a wash-out period; and studies which did not have a placebo group or proper control group.

### Data extraction and quality assessment

Extracted data from each article included the first author’s name; year of publication; study population; study design and duration of intervention; number, age, gender or sex ratio and health status in subjects; method and dose of intervention; source of samples; data of mean changes and corresponding standard deviation for PGE_2,_ TXB_2_ or LTB_4_. Risk of bias table based on Cochrane Criteria was used to assess the quality of studies in the present meta-analysis, including random sequence generation (selection bias), allocation concealment (selection bias), blinding of participants and personnel (performance bias), blinding of outcome assessment (detection bias), outcome data (attrition bias) and outcome reporting (reporting bias) [18].

### Statistical analysis

We conducted three independent meta-analysis for PGE_2,_ TXB_2_ and LTB_4_ and subgroup analysis was conducted to identify the source of heterogeneity. For parallel trials, mean changes and corresponding SDs from baseline to endpoint were collected for data analysis, data was estimated according to the previous method [19] if SDs of changes were not reported. For studies with a crossover design, mean changes between the levels of PGE_2,_ TXB_2_ and LTB_4_ and corresponding SDs at the end of two intervention periods were used in data analysis, as suggested by Cochrane handbook for Systematic Reviews of Intervention [18]. The meta-analysis was performed with Stata/SE 11.0 software (Stata Corporation, College Station, TX) by using the random effect model. Standard mean differences and 95% CIs were calculated for net changes in values with PGE_2,_ TXB_2_ and LTB_4._ Heterogeneity of treatment effects between studies was tested using the Chi-square method and I^2^ > 50% was considered to be significant. To explore the possible influence of covariates on net changes, subgroup analyses and meta-regression were conducted to evaluate the effects of dose of total marine-derived n-3 PUFA, EPA and DHA respectively, ratio of EPA/DHA, duration and design of study, body mass index (BMI) and age of subjects and type of intervention and placebo on the overall effect size. We classified studies according to duration as short term (< 12 weeks) or long term (> = 14 weeks). Region of subjects were mainly divided into four areas- Europe, America, Australia and Asia. For the following variables—age and BMI at baseline, dose of total marine-derived n-3 PUFA, EPA and DHA, ratio of EPA to DHA, we stratified studies according to their median. Meta-regression with restricted maximum likelihood estimation was performed to evaluate the potentially important covariates exerting substantial impact on heterogeneity between the trials. According to the Cochrane Handbook for Systemic Review, publication bias was examined by using funnel plots, Begg’s test and Egger’s regression test [20].

## Results

### Selection of relevant studies

**Fig 1:**
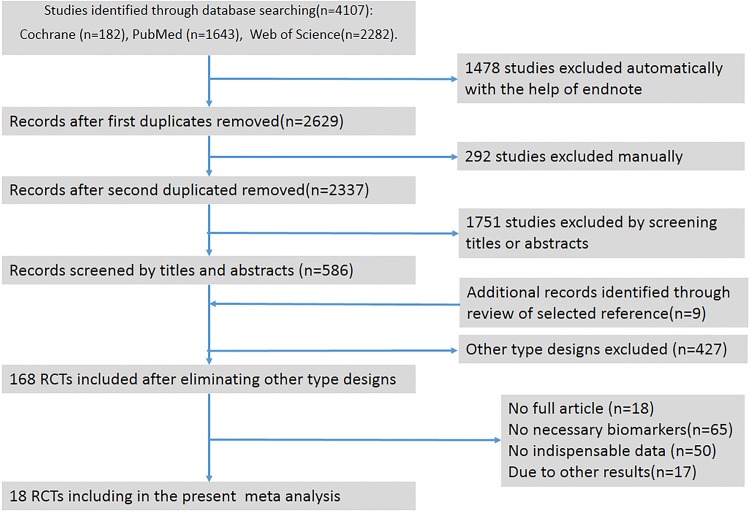
Flow diagram of studies evaluated in the review. RCT, randomized controlled trial.

The initial electronic search yielded 4107 records (1643 from PubMed, 2282 from Web of Science and 182 from Cochrane with 1770 duplicate records). Titles and abstracts were screened for 1751 citations, and 168 RCTs were obtained after eliminating other type designs. Of these, 50 RCTs were excluded because data indispensable for meta-analysis was still not available. Four studies were excluded because they only provided figures without definite information, and two studies had insufficient information (no data at baseline or endpoint). We contacteded 15 authors for data and only received replies from 4 of them, but the required data was not available. Finally, 18 studies of RCTs, including 27 study arms with 826 subjects were included in this systematic review and meta-analysis. Detailed processes of the study selection are shown in Fig 1. PRISMA Flow Diagram PRISMA and checklist required for meta-analysis are shown in S1 Fig and S1 PRISMA Checklist.

### Characteristics of the studies

Primary characteristics of the 18 studies including 27 study arms are shown in Table 1. A total of 826 subjects were randomly assigned to intervention or control groups with a completion rate of 57.23%. Of the 20 included randomized controlled trials, subjects in most studies were from Europe (n = 7), and others were from the United States (n = 4), Australia (n = 3), Brazil (n = 1), korea (n = 1), Iran (n = 1) and Canada (n = 1).

**Table 1:** Characteristics of included studies for systematic review and meta-analysis.

Studies				Subjects					Treatments				
Author	Sample	Biomarkers	Duration	No	Country	Healthy	BMI	Age	n-3	EPA	DHA	EPA	T
	type		(weeks)			status	(kg/m^2^)	(yr)	(g)	(g)	(g)	/DHA	
Andrade et al.2007	Plasma	PGE_2_	6	20	Brazil	Health	23.5	27.5	1.45	0.95	0.5	1.9	Fish oil
Tempel et al.1990	Neutrophils	LTB_4_	12	16	Netherlands	RA	UN	53	3.36	2.04	1.32	1.54	Fish oil
Ve'ricel et al.1999	Platelet	TXB_2_	6	20	France	Healthy	UN	76.5	0.18	0.03	0.15	0.2	Fish oil
Park et al.2013	Serum	PGE_2_	16	81	Korea	RA	22.4	48.4	3.255	2.09	1.165	1.79	Fish oil
Peck et al.1996 (O)	Plasma	PGE_2_	8	13	USA	Hemodialysis	25.4	50.2	UN	UN	UN	UN	Fish oil
Peck et al.1996 (S)	Plasma	PGE_2_	8	12	USA	Hemodialysis	24.4	52.2	UN	UN	UN	UN	Fish oil
Pirich et al.1999	Serum	TXB_2_	6	26	Australia	Hyper cholesterolemia	UN	57	0.356	0.216	0.14	1.54	Fish oil
Sørensen et al.1992	Serum	TXB_2_	7	34	Denmark	Pregnant	UN	29.6	2.7	UN	UN	UN	Fish oil
Singer et al.2004	Plasma	TXB_2_	24	65	Germany	Cardiac arrhyamias	25.9	43.5	0.9	0.54	0.36	1.5	Fish oil
Turini et al.1994	Neutrophils	LTB_4_	6	20	Canada	Healthy	25.3	26.5	4.5	3.3	1.2	2.75	Fish oil
Tartibian et al.2011	Plasma	PGE_2_	4	30	Iran	Healthy	24.9	30.2	0.54	0.324	0.216	1.5	Fish oil
Thusgaard et al.2009	Neutrophils	LTB_4_	12	48	Denmark	HIV-infect	24.7	45	3.36	1.84	1.52	1.21	Fish oil
Maaloe et al.2011	Neutrophils	LTB_4_	8	56	Denmark	Chronic kidney diseases	UN	59	1.44	0.864	0.576	1.5	Fish oil
Knapp et al.1989 (L)	Serum	TXB_2_	4	16	USA	Hypertension	UN	UN	3	1.8	1.2	1.5	Fish oil
Knapp et al.1989 (H)	Serum	TXB_2_	4	16	USA	Hypertension	UN	UN	15	9	6	1.5	Fish oil
Mori et al.1992	Neutrophils	LTB_4_	4	29	Australia	Peripheral vascular disease	25.4	61.9	4.6	2.8	1.8	1.56	Fish oil
Phang et al.2013	Plasma	TXB_2_	4	47	Australia	Healthy	24.6	39.6	1.2	1	0.2	5	Fish oil
Phang et al.2013 (H)	Plasma	TXB_2_	4	47	Australia	Healthy	24.6	39.6	1.2	0.2	1	0.2	Fish oil
Rees et al.2006 (YL)	MNCs	PGE_2_	12	31	UK	Healthy	24.8	24.3	1.65	1.35	0.3	4.5	EPA
Rees et al.2006 (OL)	MNCs	PGE_2_	12	22	UK	Healthy	27.6	61	1.65	1.35	0.3	4.5	EPA
Rees et al.2006 (YM)	MNCs	PGE_2_	12	31	UK	Healthy	23.5	24.8	3.3	2.7	0.6	4.5	EPA
Rees et al.2006 (OM)	MNCs	PGE_2_	12	20	UK	Healthy	28.1	60.9	3.3	2.7	0.6	4.5	EPA
Rees et al.2006 (YH)	MNCs	PGE_2_	12	31	UK	Healthy	24	24	4.95	4.05	0.9	4.5	EPA
Rees et al.2006 (OH)	MNCs	PGE_2_	12	20	UK	Healthy	27	60.1	4.95	4.05	0.9	4.5	EPA
Kremer et al.1990 (L)	Neutrophils	LTB_4_	24	26	USA	RA	UN	58.8	UN	UN	UN	UN	EPA
Kremer et al.1990 (H)	Neutrophils	LTB_4_	24	23	USA	RA	UN	58	UN	UN	UN	UN	EPA
Kremer et al.1987	Neutrophils	LTB_4_	14	26	USA	RA	UN	56.8	4.5	2.7	1.8	1.5	EPA

T, intervention in treatment group; UN, unclear; RA, rheumatoid arthritis; MNCs, mononuclear cells; PGE_2_, prostaglandin E_2_; TXB_2_, thromboxane B_2_; LTB_4_, leukotriene B_4_; L, low, H, high; YL, young and low, OL, old and low, YM, young and middle, OM, old and middle, YH, young and high, OH, old and high.

For studies that reported values of PGE_2_, TXB_2_ or LTB_4_ at more than 2 time points, only changes from baseline to endpoint were used to calculate the overall effect size [21]. For studies that included more than one intervention group, to avoid the duplication of subjects for analysis, ‘shared’ control group was split into two or more control groups with smaller sample size, which included two or more (reasonably independent) comparisons.

Knapp et al [22] compared a high dose and low dose of fish oil with safflower oil and mixed oils. The high dose arm was compared with mixed oil arm labelled Knapp L, whereas the low dose arm was compared with safflower oil arm labelled Knapp H. Kremer et al [23] included low dose, high dose and control, the control was isolated into two units and labeled Kremer L and Kremer H. Phang et al [24] compared EPA group and DHA group with Placebo group. The EPA arm was compared with Placebo arm labelled Phang E, wheras DHA arm was compared with Placebo arm labelled Phang D. Peck et al [16] compared fish oil with various control oils including olive oil and safflower oil, the fish oil arm was seperated into two small arms labelled Peck O and Peck S respectively. There were eight groups in the study reported by Rees et al [25], six study arms were labelled Rees (YL), Rees (OL), Rees (YM), Rees (OM), Rees (YH), Rees (OH) respectively according to age (Y-young, O-old) and dose (L-low, M-moderate, H-high).

### Quality of studies

**Fig 2:**
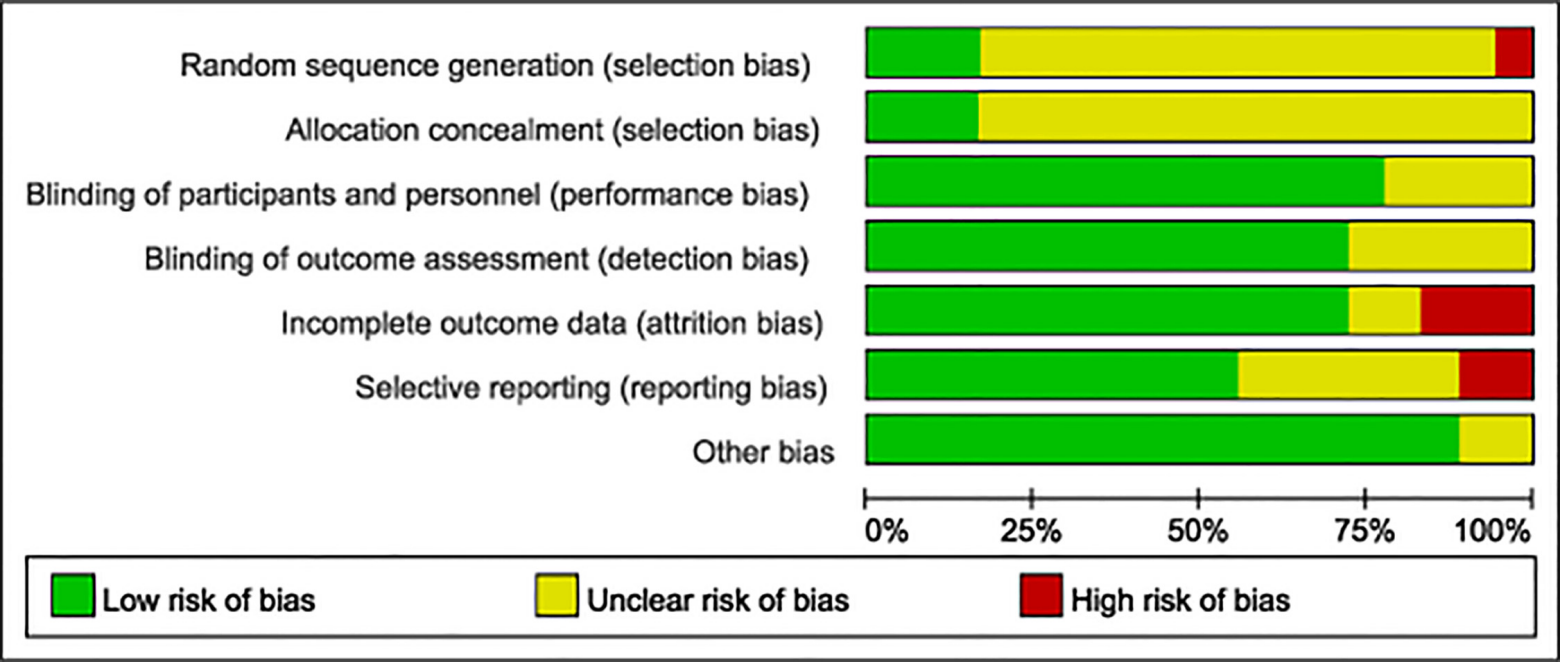
Risk-of-bias graph. The review of judgments of authors about each risk-of-bias item is presented as percentages across all included studies.

**Fig 3:**
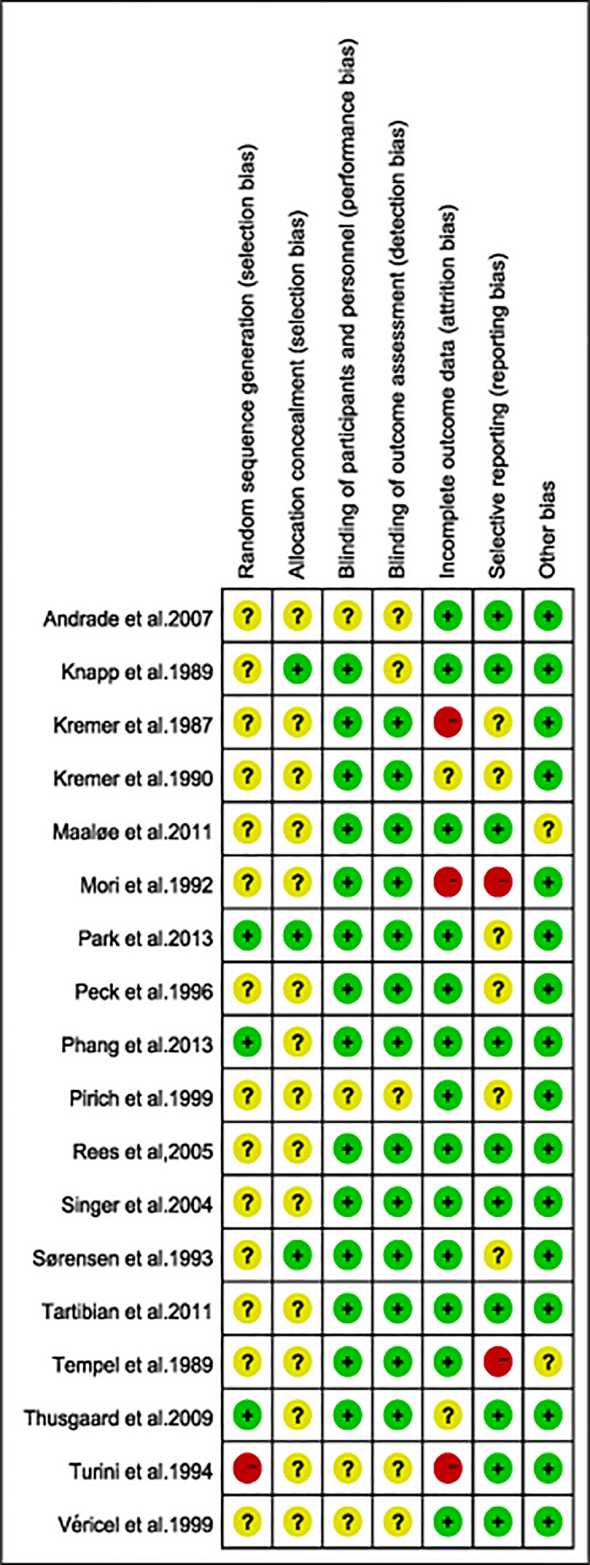
Risk of bias summary. Review author’s judgements about each risk of bias item for each included study.

None of the included trials reported any significant differences in characteristics of subjects in the treatment or control arm. Allocation concealment was adequate in 5 studies and unclear in others. Sixteen studies were double-blind design, one study [22] only masked paticipants, and five studies were unclear. Risk-of-bias graph and risk of bias summary are shown in Figs 2 and 3.

### Effect of marine-derived n-3 PUFA supplementation on PGE_2_

Five studies reported the effect of marine-derived n-3 PUFA on PGE_2_ [16, 25–28], among which two studies [26, 28] included in the meta-analysis showed a significant reduction on PGE_2_ in plasma of healthy subjects with the supplementation of marine-derived n-3 PUFA compared with placebo supplementation (SMD:-1.27; 95% CI: -1.90,-0.63).

### Effect of marine-derived n-3 PUFA supplementation on TXB_2_

**Fig 4:**
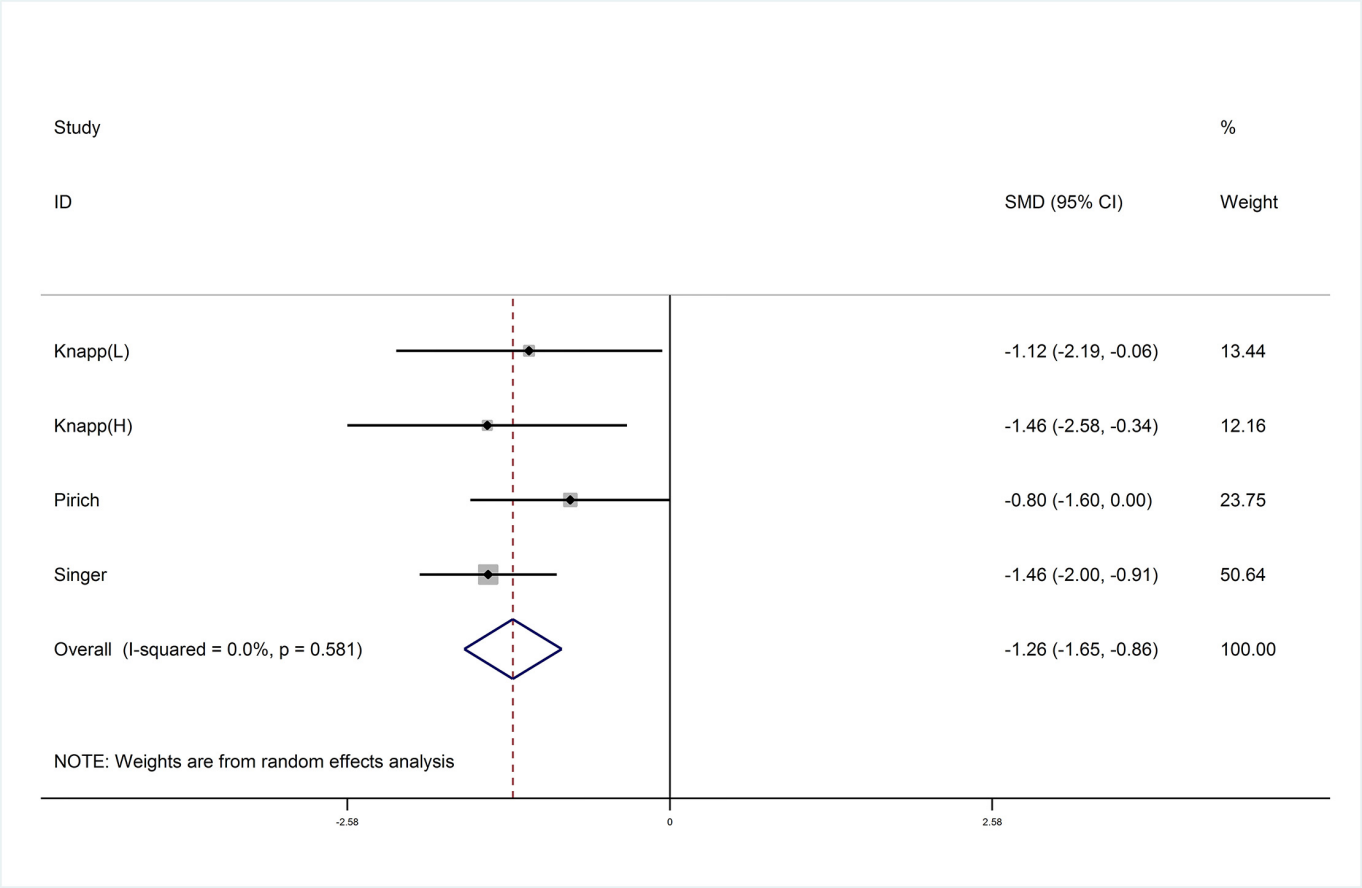
Pooled effect size of marine-derived PUFA supplementation on TXB_2_.in subjects with high risk of CVD. CVD, cardiovascular disease. SMD, standard mean difference.

Six studies [21, 22, 24, 29–31] were included in this systematic review and meta-analysis, and data from three studies [22, 30–31] with four arms were pooled to demonstrate that marine-derived n-3 PUFA showed a siginificant difference in reducing TXB_2_ in subjects with high risk of CVD compared with placebo (SMD:-1.26; 95% CI: -1.65, -0.86; I^2^ = 0.0%) (Fig 4).

### Effect of marine-derived n-3 PUFA supplementation on LTB_4_

**Fig 5:**
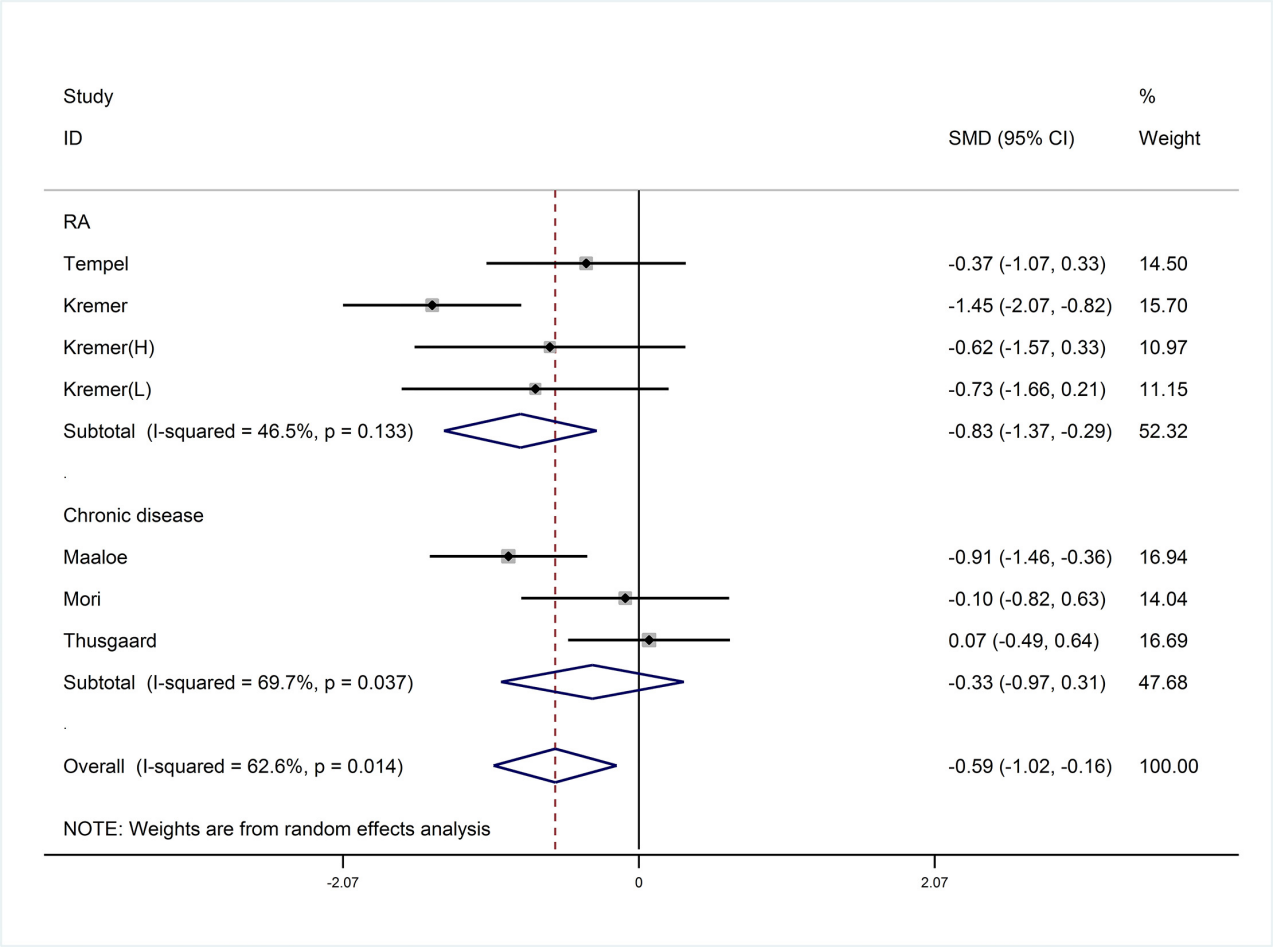
Pooled effect size of marine-derived n-3 PUFA supplementation on LTB_4_ in unhealthy subjects including RA and Chronic diseases patients. RA, rheumatoid arthritis. SMD, standard mean difference.

Six studies [23, 32–36] with seven study arms reported the effect of marine-derived n-3 PUFA on LTB_4_ in unhealthy subjects and the other one [37] reported in healthy subjects. Marine-derived n-3 PUFA was more effective in reducing the concentration of LTB_4_ in neutrophils of unhealthy subjects compared with placebo (SMD:-0.59; 95% CI: -1.02, -0.16; I^2^ = 62.6%) (Fig 5).

### Subgroup analysis and meta-regression

Subgroup analyses and meta-regression analyses were conducted to evaluate the effects of the confounding factors on primary outcomes of marine-derived n-3 PUFA on LTB_4_ for each potential variable including dose of total marine-derived n-3 PUFA, EPA and DHA, ratio of EPA to DHA, duration and design of study, body mass index (BMI) and age of subjects and type of intervention and placebo. Full details are shown in Table 2. Subgroup analyses showed a more significant reduction on LTB_4_ in RA patients with supplementation of marine-derived n-3 PUFA (SMD: -0.83; 95% CI: -1.37, -0.29), but not in non-autoimmune chronic disease patients (SMD: -0.33; 95% CI: -0.97, 0.31) (Fig 5). No significant reduction on LTB4 was found when the duration of intervention with marine-derived n-3 PUFA was less than 14 weeks (SMD: -0.34; 95% CI: -0.81, 0.13) (Table 2). There was no significant difference in serum (SMD: -1.05; 95% CI: -1.61, -0.49) and plasma (SMD: -1.46; 95% CI: -2.00, -0.91) concerning the effect of marine-derived n-3 PUFA on TXB_2_. The use of anti-inflammatories failed to significantly change the results for the effect of marine-derived n-3 PUFA on LTB_4_ (P = 0.286).

**Table 2:** Subgroup analysis for the effect of marine-derived n-3 PUFAs on LTB_4_ in unhealthy subjects (subjects with non-autoimmune chronic diseases or auto-immune diseases).

Subgroup analysis	LTB_4_				
	N	SMD (95%CI)	Heterogeneity		P_2_
			I^2^ (%)	P_1_	
Overall	7	-0.59 (-1.12, -0.25)	62.6	0.014	
Sex ratio(male/female)					0.286
<0.5	3	-0.33 (-0.97, 0.31)	69.7	0.037	
> = 0.5	4	-0.83 (-1.37, -0.29)	46.5	0.133	
Age					0.965
<median	3	-0.58 (-1.50, 0.34)	84.4	0.002	
> = median	4	-0.63 (-1.01, -0.25)	3.80	0.374	
Health status					0.163
Chronic diseases	3	-0.33 (-0.97, 0.31)	69.7	0.037	
Rheumatoid arthritis	4	-0.83 (-1.37, -0.29)	46.5	0.133	
Duration					0.138
<14weeks	4	-0.34 (-0.81, 0.13)	54.6	0.086	
> = 14 weeks	3	-1.04 (-1.59, -0.48)	27.8	0250	
Daily dose of total n-3 PUFA(g)					0.987
<median	2	-0.69 (-1.21, -0.16)	30.0	0.232	
> = median	3	-0.49 (-1.46, 0.48)	85.6	0.001	
NR	2	-0.68 (-1.34, -0.06)	0	0.880	
daily dose of EPA(g)					0.679
<median	2	-0.42 (-1.39, 0.54)	83.2	0.015	
> = median	3	-0.65 (-1.49, 0.18)	77.8	0.011	
NR	2	-0.68 (-1.34, -0.01)	0	0.880	
daily dose of DHA(g)					0.987
<median	2	-0.69 (-1.21, -0.16)	30.0	0.232	
> = median	3	-0.49 (-1.46, 0.48)	85.6	0.001	
NR	2	-0.68 (-1.34, -0.01)	0	0.880	
Ratio of EPA/DHA					0.765
< 1.55	3	-0.76 (-1.62, 0.10)	84.8	0.001	
> = 1.55	2	-0.24 (-0.74, 0.27)	0	0.594	
NR	2	-0.68 (-1.34, -0.01)	0	0.880	
Region					0.927
Europe	3	-0.41 (-1.01, -0.19)	66.6	0.500	
America	3	-1.04 (-1.59, -0.48)	27.8	0.250	
Australia	1	-0.10 (-0.82, 0.63)	NA	NA	

N, number of included studies (or comparisons); NR, not reported; NA, not associated with this item; P_1_ value for heterogeneity within each subgroup; P_2_ value for heterogeneity between subgroups with meta-regression analysis.

### Sensitivity analysis

**Fig 6:**
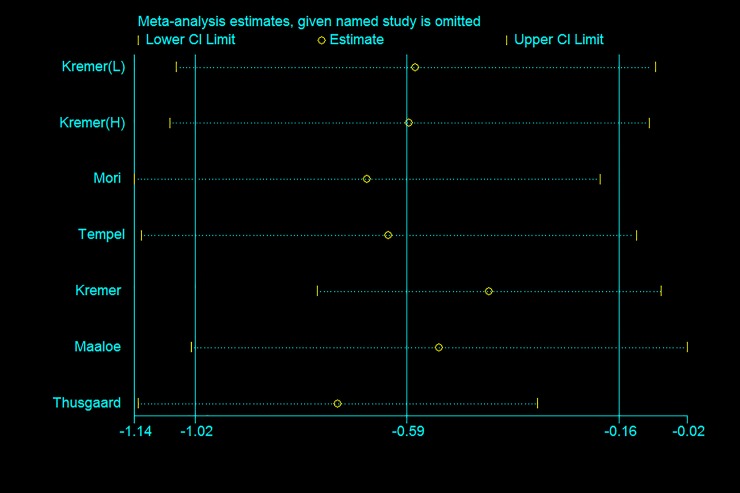
Sensitive analysis with pseudo 95% confidence limits for studies with LTB_4._

We also conducted sensitivity analyses to explore the source of heterogeneity. Sensitivity analysis was performed by studying whether effect size changes significantly after excluding special trials one by one. Some trials were not consistent with others in terms of intervention method, measurement method, samples and volunteers. Rees et al [25] detected PGE_2_ concentration in mononuclear cell; special volunteers were recruited such as elite swimmers [28] and pregnant women [21]; Radioimmunoassay[16, 22, 30] and ELISA [24–29, 31] were used to determine the concentrations of PGE_2_ and LTB_4_. Moreover, the studies by Mori et al [33], Pirich et al [30] and Turini et al [37] had low quality and high risk bias. However, the results of the sensitivity analysis on PGE_2_, TXB_2_ and LTB_4_ (Fig 6) showed that the pooled effects remained non-significant when analyses were limited to high-quality trials and conducted after excluding special trials.

In the study by Knapp [22], the high dose arm was compared with mixed oil arm labelled Knapp L, whereas the low dose arm was compared with safflower oil arm labelled Knapp H. The pooled effects remained non-significant when we compared the high dose arm with safflower oil and low dose arm with mixed oil arm.

### Publication bias

**Fig 7:**
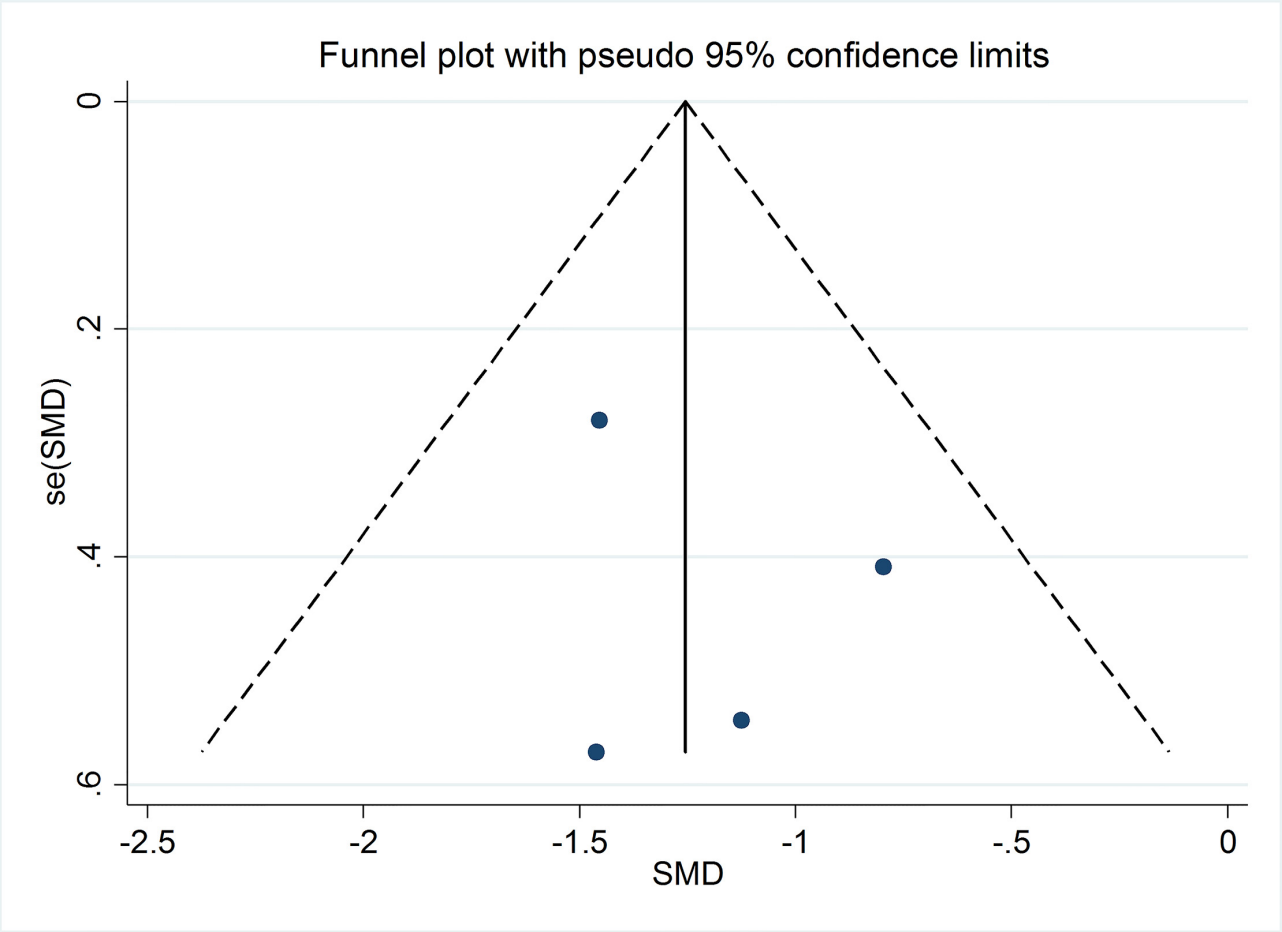
Funnel plot with pseudo 95% confidence limits for studies with TXB_2._

**Fig 8:**
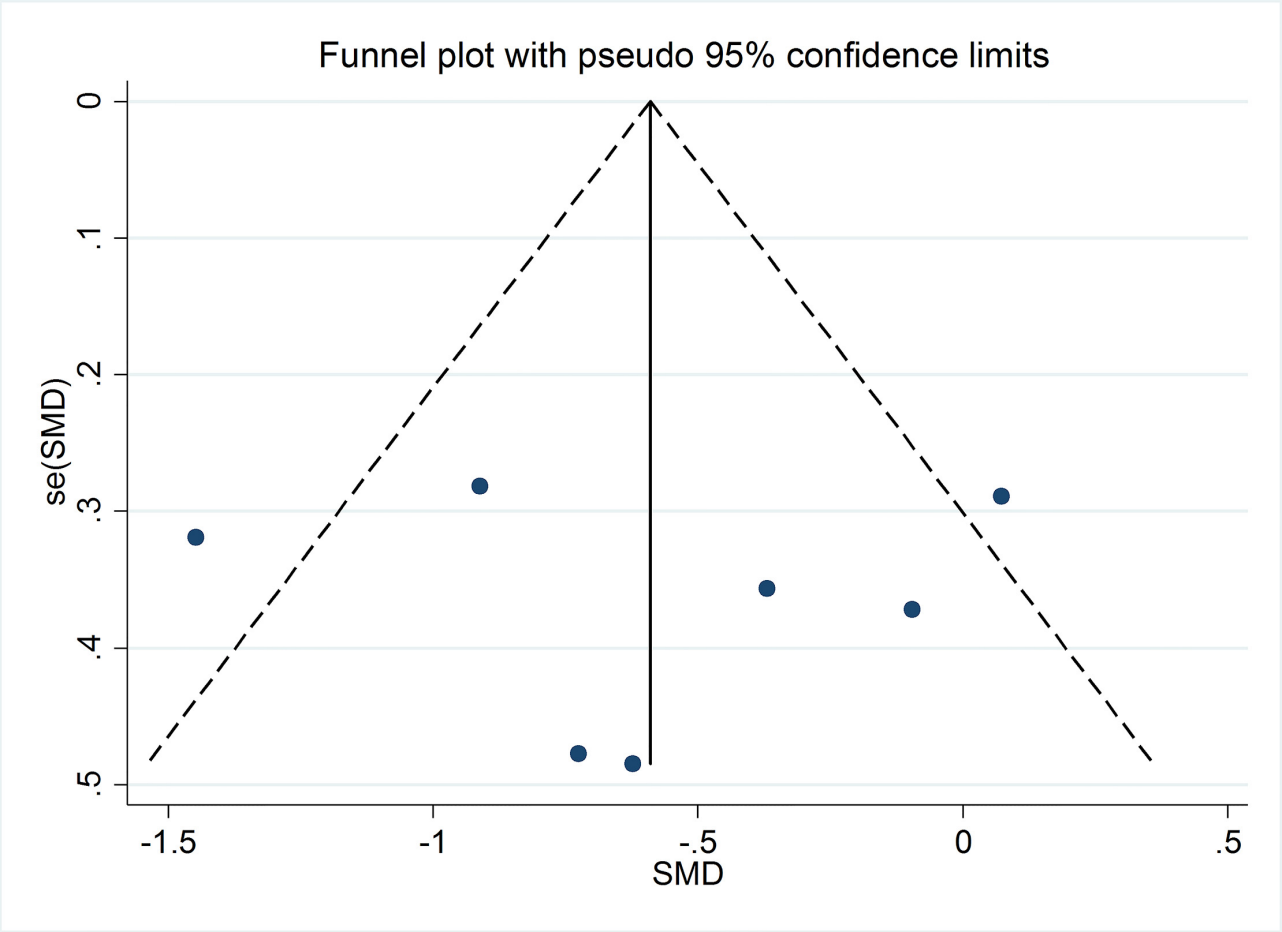
Funnel plot with pseudo 95% confidence limits for studies with LTB_4._

On the basis of funnel plots (Figs 7 and 8) and Egger’s test, no significant publication bias was shown in the meta-analysis of TXB_2_ in serum or plasma of subjects with high risk of CVD (P = 0.59) and LTB_4_ in neutrophils of unhelthy subjects (P = 0.60) with supplementation of marine-derived n-3 PUFA.

## Discussion

The results of this meta-analysis supported the effect of marine-derived n-3 PUFA on suppressing TXB_2_ in subjects with high risk of CVD and LTB_4_ in neutrophils in unhealthy subjects. TXB_2_ can induce vasoconstriction and promote aggregation of platelets as well as adhesiveness of PMNs, and LTB_4_ is involved in modulating the intensity and duration of inflammatory responses. On one hand, n-3 PUFAs, especially marine-derived n-3 PUFAs, directly inhibit AA metabolism to produce eicosanoids by replacing AA as the substrate, and indirectly alter the expression of inflammatory genes through effects on transcription factor genes [12]. On the other hand, marine-derived n-3 PUFAs, especially EPA, can also act as a substrate catalyzed by 5-lipoxygenase enzymes and cyclooxygenase enzymes to generate several eicosanoids. These eicosanoids, such as LTB_5_ and LTE_5_, have slightly different structures and less potent pro-inflammatory function compared with those formed from AA [38]. Furthermore, EPA and DHA can also generate a novel genus of mediators, termed specialized pro-resolving mediators.

Subgroup analysis results showed a significant effect on LTB_4_ in RA patients in neutrophils with supplementation of marine-derived n-3 PUFA, Park et al [27] also found that n-3 PUFA supplementation significantly decreased LTB_4_ levels in serum of RA patients who weighed more than 55kg. For subjects with chronic diseases, the function of marine-derived n-3 PUFA did not reach statistical significance. However, PGE_2_ was significantly increased in hemodialysis patients compared with control group following supplementation of marine-derived n-3 PUFA [16], and a slightly but not significant increase was also found in RA patients [27]. In addition, marine-derived n-3 PUFA did not result in significantly decreased formation of plasma PGE_2_ in mildly hypertriacylglycerolemic subjects [39]. A hypothesis was put forward to explain the different results between PGE_2_ and LTB_4_: a decrease in proteinuria and an improvement in glomerular filtration rate was reported [40], which may be relevant to the function of n-3 PUFA on immunoglobulin A nephropathy. Increased DHA and EPA could result in the production of inactive LTB_5_ along with decreased synthesis of the inflammatory LTB_4_. These eicosanoids were produced through different pathways, LTB_4_ was generated through lipoxygenase pathway with 5-lipoxygenases enzymes, while PGE_2_ and TXA_2_ /TXB_2_ were from cyclo-oxygenase (COX) pathway. So LTB_5_ blocked the lipoxygenase pathway and arachidonic acid was metabolized to produce PGE_2_ through the cyclooxygenase pathway, which led to a decrease in LTB_4_ and corresponding increase in PGE_2_.

However, for another product of the cyclooxygenase pathway, TXB_2_ showed a reduction with supplementation of marine-derived n-3 PUFA in populations with high risk of CVD. Mehta et al. [41] found that EPA supplementation had no effect on concentrations of PGE_2_ in Barrett’s mucosa. This study also suggested no significant alteration in AA and increase in phospholipase A_2_ activity. Hishinuma et al. [42] demonstrated that an increased availability of AA resulted in an increase in 2-series eicosanoid production in murine mast cells, and the predominant COX2 pathway products were PGE_2_ > PGF_2α_ > TXB_2_ > 6-keto-PGF_1α_ [43, 44] when AA is in abundance. Therefore, it may lead to no significant change in PGE_2_ and a reduction in TXB_2_ concentration.

An animal experiment by Kang et al [45] examined this issue from another aspect. They measured the inflammatory indicators in wild type (WT) mice and fat-1 mice (a kind of transgenic mice rich in endogenous n-3 PUFA), both of which were induced to have colitis. Results showed that in colon tissue, no significant differences in the content of AA, LTB_4_ and PGE_2_ were found, but there was a remarkable increase in the amounts of EPA and DHA as well as their potent bioactive products including resolvins and protectins (RvE1, RvD3, and PD1/NPD1), and the less potent products including LTB_5_ and PGE_3_. The author assumed that marine-derived n-3 PUFA exerts anti-inflammatory functions by the potent bioactive rather than AA-derived eicosanoids. This hypothesis could explain why there was no difference in PGE_2_ levels after marine-derived n-3 PUFA supplementation, but this conflicted with our results for TXB_2_ and LTB_4_. The inconsistency may be most likely due to the difference in the ratio of n-3/n-6 PUFA. In the study by Kang et al [45], the n-3/n-6 ratio was so high in fat-1 mice that it could supply limited AA as substrate to rise PGE_2_, TXB_2_ and LTB_4_. More animal experiments are needed to explore the mechanism of how marine-derived n-3 PUFA exerts its effect on inflammation.

In healthy subjects, marine-derived n-3 PUFA supplementation had no significant effect on TXB_2_ [21, 24], which is consistent with the result from Murphy et al. [46], who found that levels of TXB_2_ tended to decrease with n-3 PUFA supplementation in both groups, but this did not reach statistical significance. It was most likely due to the low production of eicosanoids at baseline in healthy subjects. However, Andrade et al. found swimming athletes who had supplementation of marine-derived n-3 PUFA showed decreased PGE_2_ in plasma [28], which was also confirmed in common healthy subjects reported by Tartibian et al [26], and EPA also led to a significant reduction in the levels of PGE_2_ in mononuclear cell (MNC) [25] and skin unexposed to ultraviolet radiation [47]. The different results between PGE_2_ and TXB_2_ may result from the different dose of n-3 PUFA. Individuals with a higher concentrations of EPA and DHA into erythrocyte membranes also showed the greatest reduction in erythrocyte AA content [48]. A substantial increase in the EPA content of MNC phospholipids (> 4-fold) and a corresponding decrease in AA (25%) was required to affect PGE_2_ production [25].

We only included studies for meta-analysis where PGE_2_ were measured in plasma of healthy subjects, and TXB_2_ were tested in in serum/plasma in subjects with high risk of CVD (Fig 4) and LTB_4_ were measured in neutrophils of unhealthy subjects (Fig 5). Others were only included for systematic review and reported in discussion. Additionally, LTB_4_ was detemined by HPLC, while PGE_2_ and TXB_2_ were measured by radioimmunoassay or ELISA. We conducted SMD as the estimated effect size and random effects model was used for meta-analysis.

We first utilized pooled data to quantitatively assess the effect of marine-derived n-3 PUFA on AA-derived major eicosanoids (PGE_2,_ TXA_2_/TXB_2_ and LTB_4_), and subgroup analyses were conducted to evaluate the effects of confounding factors on overall effect size of LTB_4_ in unhealthy subjects. In addition, we discovered the effects of marine-derived n-3 PUFA on PGE_2_ and TXB_2_ were different, and future studies can recruit volunteers with different health status to explore the effect of n-3 PUFA on eicosanoids. Lastly, no significant publication bias was shown in the meta-analysis according to the results of funnel plots and Egger’s test.

There are several limitations in the present study. First, to explore the potential influence on effect size by several confounding factors (such as age of subjects and dose of n-3 PUFA), ‘shared’ control group was split into two or more control groups to couple the intervention group according to the method by Cochrane handbook [18]. Studies included in one meta-analysis were so limited that all trials in a subgroup were likely to be from one study in subsequent subgroup analysis. Secondly, the placebo in control group showed variety including olive oil, safflower, mineral oil, corn oil and others, and subgroup analysis by the type of placebo couldn’t be performed for too few trials in each subgroup. Lastly, dose-response of the effect of marine-derived n-3 PUFA on major eicosanoids was also not conducted due to limited trials. Therefore, more well-controlled RCTs to investigate the effect of marine-derived n-3 PUFA on major eicosanoids are needed to solve the problems above.

## Conclusions

In conclusion, this systematic review and meta-analysis provided evidence that marine-derived n-3 PUFA had a significant benefit on the reduction of TXB_2_ content in subjects with high risk of CVD and LTB_4_ concentrations in unhealthy subjects. Subgroup analyses showed that marine-derived n-3 PUFA significantly decreased LTB_4_ in RA patients, but not in non-autoimmune chronic disease patients, and duration of intervention with marine-derived n-3 PUFA also affected the overall effect size. High quality RCTs are needed to explore the effects of marine-derived n-3 PUFA on different eicosanoids in subjects with different health status.

## Supporting Information

S1 PRISMA Checklist PRISMA 2009 checklist. (DOC)

S1 Fig PRISMA flow diagram of the search and study selection process. (DOC)
