# Supplementary material for: Effect of Marine-Derived n-3 Polyunsaturated Fatty Acids on Major Eicosanoids: A Systematic Review and Meta-Analysis from 18 Randomized Controlled Trials
Source: PLoS One. 2016 Jan 25;11(1):e0147351. doi: 10.1371/journal.pone.0147351 (PMC4726565; doi:10.1371/journal.pone.0147351)
Supplement: S1 Fig — (DOC) [file pone.0147351.s002.doc]

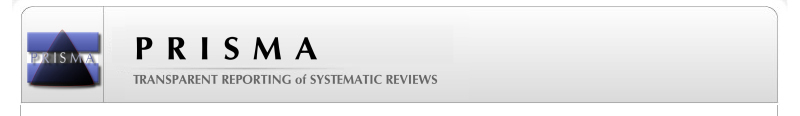
**PRISMA 2009 Flow Diagram**

**Screening**

**Included**

**Eligibility**

**Identification**

Records identified through database searching
(n = 4107 )

Additional records identified through other sources
(n = 9 )

Records after duplicates removed
(n = 2337 )

Records screened
(n =586 )

Records excluded
(n = 1751 )

Full-text articles assessed for eligibility
(n = 168 )

Full-text articles excluded, with reasons
(n =150 )

Studies included in qualitative synthesis
(n =18 )

Studies included in quantitative synthesis (meta-analysis)
(n =18 )
